# Using Resistance-Band Tests to Evaluate Trunk Muscle Strength in Chronic Low Back Pain: A Test–Retest Reliability Study

**DOI:** 10.3390/s24134131

**Published:** 2024-06-25

**Authors:** Francisco Franco-López, Krzysztof Durkalec-Michalski, Jesús Díaz-Morón, Enrique Higueras-Liébana, Alejandro Hernández-Belmonte, Javier Courel-Ibáñez

**Affiliations:** 1 Department of Physical Activity and Sport, Faculty of Sport Sciences, University of Murcia, 30720 Murcia, Spain; francisco.francol@um.es (F.F.-L.); enriquehiglie@gmail.com (E.H.-L.); alejandro.hernandez7@um.es (A.H.-B.); 2 Department of Sports Dietetics, Poznan University of Physical Education, 61-871 Poznan, Poland; durkalec-michalski@awf.poznan.pl; 3 SANO Sport Center, el Toyo, 04131 Almería, Spain; jesus.diaz.pt@gmail.com; 4 Department of Physical Education and Sports, Faculty of Education and Sport Sciences, University of Granada, 52005 Melilla, Spain

**Keywords:** musculoskeletal pain, physical fitness, spine, resistance training, elastic bands

## Abstract

Exercise is a front-line intervention to increase functional capacity and reduce pain and disability in people with low strength levels or disorders. However, there is a lack of validated field-based tests to check the initial status and, more importantly, to control the process and make tailored adjustments in load, intensity, and recovery. We aimed to determine the test–retest reliability of a submaximal, resistance-band test to evaluate the strength of the trunk stability muscles using a portable force sensor in middle-aged adults (48 ± 13 years) with medically diagnosed chronic low back pain and healthy peers (*n* = 35). Participants completed two submaximal progressive tests of two resistance-band exercises (unilateral row and Pallof press), consisting of 5 s maintained contraction, progressively increasing the load. The test stopped when deviation from the initial position by compensation movements occurred. Trunk muscle strength (CORE muscles) was monitored in real time using a portable force sensor (strain gauge). Results revealed that both tests were highly reliable (intra-class correlation [ICC] > 0.901) and presented low errors and coefficients of variation (CV) in both groups. In particular, people with low back pain had errors of 14–19 N (CV = 9–12%) in the unilateral row test and 13–19 N (CV = 8–12%) in the Pallof press. No discomfort or pain was reported during or after the tests. These two easy-to-use and technology-based tests result in a reliable and objective screening tool to evaluate the strength and trunk stability in middle-aged adults with chronic low back pain, considering an error of measurement < 20 N. This contribution may have an impact on improving the individualization and control of rehabilitation or physical training in people with lumbar injuries or disorders.

## 1. Introduction

Low back pain is the world’s leading cause of disability, surpassing depression, diabetes, and coronary heart disease [1]. It is estimated that 80% of the world’s population has suffered from low back pain at some stage in life [2]. Indeed, low back pain is a problem not only for individuals but also for the global health and economy systems by increasing the associated care costs and reducing labor productivity [3]. Low back pain can evolve into degenerative lumbar spine diseases and become chronic if left untreated, rehabilitation fails or complications appear, which substantially reduce people’s quality of life and likely require surgery. Lumbar arthrodesis (fusion of two or more discs) and discectomy (removal of the degenerative discs) are two of the most common surgical techniques to treat chronic low back pain [4]. Although minimal surgery interventions are available, it is common for people to undergo invasive techniques, which leads to physical disability that can worsen over the years and may require a reoperation [5]. Hence, following an effective rehabilitation process is vital to reduce risks, comorbidities, and disability in people with chronic low back pain.

Exercise training is a front-line treatment to increase functional capacity and reduce pain and disability in middle-aged and older adults with chronic low back pain [6,7]. Even a minimal dose approach (≤60 min, 2 d·wk^−1^), using uncomplicated equipment/methods (e.g., elastic bands), is proven to produce numerous health benefits [8], which contribute to reducing risk factors for low back pain in middle-aged adults, such as excessive visceral adipose tissue or deficient skeletal muscle mass (sarcopenia) [9]. Exercise interventions focused on the strengthening of the stability trunk muscles that surround and protect the spine (CORE) [10] have shown high effectiveness in treating lumbar disorders [11,12,13,14,15,16]. The unilateral row and the Pallof press are two commonly used exercises to strengthen the trunk muscles [17,18,19]. These exercises are usually performed using resistance bands as they are a more easy-to-use alternative to machines or free weights [18,20]. While more practical, one challenge when prescribing resistance-bands training is the lack of validated field-based tests to check the initial status and, more importantly, to control the process and make tailored adjustments in load, intensity, and recovery to maximize strength adaptations [21,22]. This is critical in people with musculoskeletal injuries or disorders to minimize the hazards of overload or discomfort resulting from a bad exercise intensity prescription.

Advances in technology allow nowadays the use of portable sensors to collect data in real time and obtain information on the neuronal and neuromuscular determinants of performance, assisting in a better design and control of exercise plans [23,24,25]. In addition, these systems may increase adherence to the training program by providing instant feedback on the screen or smartphone, while ensuring that the volume and intensity aims are being accomplished [26]. Although these high-quality training methods are well-established in sports performance, their inclusion in clinical settings is still limited. Besides assisting in the training and decision-making process, the use of real-time data and visual feedback would contribute to patient education and the development of a patient-driven rehabilitation protocol [27].

To improve the individualization and control of rehabilitation programs in people with low strength levels (e.g., oncology population, older adults) or disorders (e.g., spinal injuries), the current study aimed to determine the test–retest reliability of two submaximal (5 s maintained contraction), resistance-band tests to evaluate the strength of the trunk stability muscles using a portable force sensor in middle-aged adults with chronic low back pain.

## 2. Materials and Methods

### 2.1. Experimental Design

This is an observational study examining the test–retest reliability of two elastic band tests specifically designed for trunk strength assessment in people with chronic low back pain. The study involved people with chronic low back pain (*n* = 18) and healthy peers (*n* = 17) to identify the potential variabilities in the distribution of errors related to the condition. Participants completed two submaximal strength tests (unilateral rows and Pallof press), consisting of a 5 s maintained contraction using resistance bands, in two testing sessions (test–retests) with 72 h rest to avoid the effects of residual fatigue or soreness [28,29]. Tests’ order and hands (dominant vs. non-dominant) were randomized in the test and reproduced in the retest. Before each session, participants performed the same specific warm-up to activate the muscles and set the initial load. All participants were able to complete the tests; however, they did not follow any familiarization session. Force was monitored in real time using a portable strain gauge anchored to the wall (i.e., the further from the wall, the greater the resistance-band tension and the greater the force sustained during the test).

### 2.2. Participants

Ten men and eight women with medically diagnosed chronic low back pain (*n* = 18; age 49 ± 12 years; body mass 81.8 ± 11.2 kg) were recruited from a Hospital Neurosurgery Unit in Murcia (Spain) and provided medical consent to take part in the study. Inclusion criteria were (i) people older than 18 years with symptoms of persistent low back pain over 3 months, (ii) and/or gait claudication with a diagnosis of a herniated disc, (iii) and/or canal stenosis, (iv) and/or lumbar segmental instability, (v) and/or have undergone surgery for instrumented lumbar arthrodesis. Exclusion criteria were (i) combined cervical surgery or thoracic myelopathy, (ii) inability to properly follow the isometric rowing test, and (iii) failure to perform activities in daily life due to comorbidities (stroke, Parkinson’s, psychotic disorders, or other degenerative orthopedic diseases). A convenience sample of healthy peers of 12 men and 5 women without low back pain or physical injury were recruited and volunteered to participate (*n* = 17; age 47 ± 13 years; body mass 78.0 ± 17.2 kg). All participants were informed of the procedures and objectives of the study and later signed the informed consent, which was approved by the Ethics Committee of the University of Murcia (ID:2754/2020) and conducted in accordance with the Declaration of Helsinki.

### 2.3. Testing Procedures

Measurements took place in the Unit of Physical Training from the Hospital, under medical supervision and standardized conditions (temperature ~22 °C, humidity ~50%). Tests were performed individually by the same researchers. Participants attended the facilities at their convenience during the morning or the afternoon, and the time of the day was replicated in both testing sessions. A portable strain gauge with incorporated software (Chronojump 2.43, Barcelona, Spain) sampling at 80 Hz was secured to the wall at a 1-m height. The strain gauge was calibrated before each session (Eleiko, Halmstad, Sweden) according to the manufacturer’s specifications. Two resistance bands (CiSport, Zaragoza, Spain), either yellow (25 kg, less stiff) or purple (35 kg, stiffer), were attached to the strain gauge. The load (tension) provided by each band was measured before evaluation by recording the increase in N every 30 cm from the wall. These reference values were used to determine the resistance band and starting distance to be used by each participant according to their physical condition. Upon arrival at the facilities, the body mass (bm) was assessed using a scale (Tanita SC331S, Tanita Corp., Tokyo, Japan).

A handle specifically designed to push and pull exercises was attached using a safety carabiner and used as a grip to perform the exercises. Warm-up consists of three sets of five repetitions of each exercise with progressive increasing tension to identify the initial load. After the warm-up, participants completed the submaximal progressive tests, consisting of 5 s maintained contraction, progressively increasing the load (i.e., the distance from the wall increased one step, ~30 cm), with 30 s rest intervals between [14]. The test stopped when one of these conditions occurred: (1) loss of >10 N within the last 3 s and (2) deviation from the initial position by compensation movements (hip rotation, trunk extension, elbow flexion). Participants completed the tests with the dominant and non-dominant hands. Volunteers were asked to report if they felt discomfort and/or pain after each execution.

**Figure 1:**
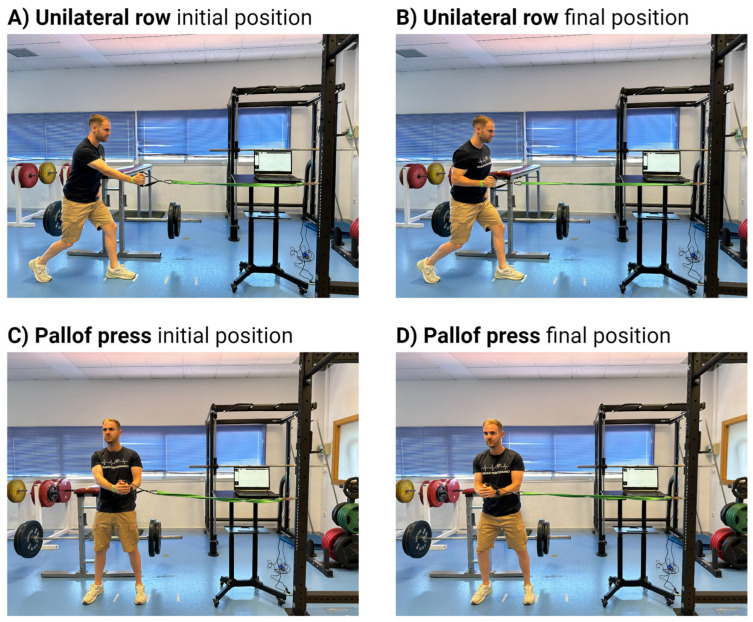
Initial and final positions for the unilateral row (**A**,**B**) and Pallof press (**C**,**D**) submaximal tests.

Participants completed these procedures in two anti-rotation muscle strength exercises: the unilateral row and the Pallof press (Figure 1). In the unilateral row [30], participants started grasping the resistance band with one hand, moving away (backward) from the wall to give tension to the resistance band, and pulling the band bringing the elbow in line with the hip. The foot of the body side that performed the pull remained behind, while the foot on the opposite side was brought forward to provide stability. In the Pallof press [19], participants had to grasp the resistance band with both hands and keep it at chest level, move away (sideways) from the place where the resistance band was anchored to increase its tension, and bring their hands forward in a horizontal plane avoiding torso twisting. For right-handed participants, the dominant hand was considered when the right hand was the closest to the wall. Force data were collected in absolute values (N) and computed relative to body mass (N·bm^−1^). Mean values for each execution were used for the analyses.

### 2.4. Statistical Analysis

D’Agostino–Pearson tests were used to test normality at each testing condition. Linear regression analysis was conducted to examine the relationship between the tension and distance to the wall for each resistance band and identify reference values. The test–retest reliability of each evaluation test was determined by the intraclass correlation (ICC) and the standard error of the measurement (SEM). A two-way mixed-effects, absolute agreement model in ICC was conducted according to guidelines for test–retest reliability [31]. The SEM was calculated from the square root of the mean square error term in a repeated-measures ANOVA to determine the measurement error and between-participant variability [32]. This statistic was expressed both in absolute (N) and relative terms as a coefficient of variation (CV = 100 SEM/mean). Criteria for acceptable reliability were set for very high (CV ≤ 5%, ICC ≥ 0.90), high (CV ≤ 10%, ICC > 0.90), and moderate (CV ≤ 15%, ICC > 0.80) [31]. Student’s *t*-test for paired samples was performed to identify significant differences (*p* < 0.05) between the test and retest conditions. Effect size (ES) was calculated to estimate the magnitude of the differences using the Hedges’ g and interpreted as low (0.20), medium (0.50), and high (0.80) [33]. Calculations were made using an Excel sheet and GraphPad Prism version 8.0.0 for Windows (GraphPad Software, San Diego, CA, USA).

## 3. Results

**Figure 2:**
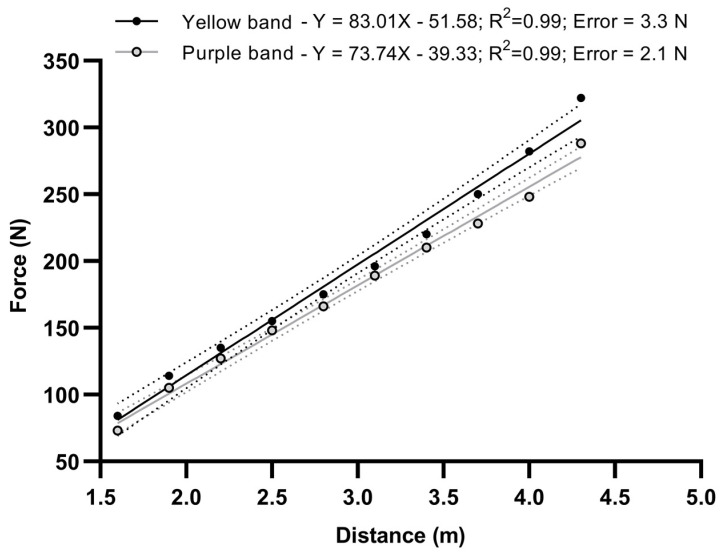
Linear relationship between the resistance provided by each resistance band in N and the distance from the wall.

All testing conditions showed a normal distribution (D’Agostino–Pearson tests *p* < 0.319). All participants completed both testing sessions reporting no discomfort or pain during or after the executions. Participants completed on average 3 ± 1 executions per exercise to reach their submaximal performance. Initial loads ranged from 1.6 to 2.7 N·bm^−1^ in women and 1.7 to 3.0 N·bm^−1^ in men. The relationship between the tension and distance from the wall is shown in Figure 2. For every 30 cm, the tension increased from 20 to 40 N. The yellow band offered 10 to 35 N higher tension compared to the purple.

**Figure 3:**
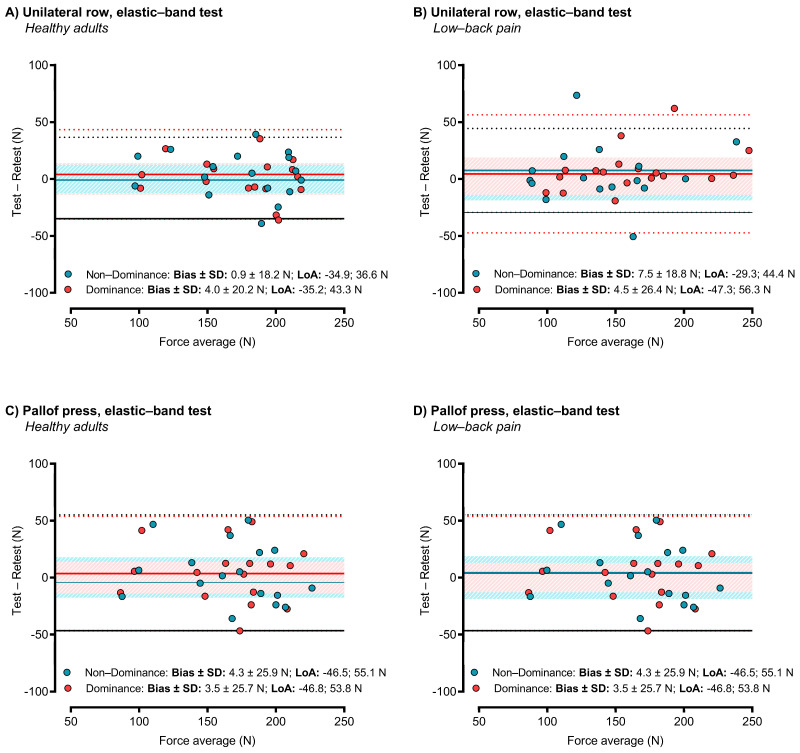
Bland–Altman plots showing the test–retest reliability of the resistance-band tests. The shaded area is the standard error of measurement (SEM). The dotted lines are the 95% limits of agreement (LoA). The straight lines are the bias.

Results of the test–retest reliability are presented in Table 1. Both tests were highly reliable with low errors and high correlation coefficients, both with dominant hands/sides (Rowing: SEM = 18.8 N, CV = 12.0%, ICC > 0.901; Pallof press: SEM = 12.9, CV = 8.4%, ICC = 0.960) and non-dominant hands/sides (Rowing: SEM = 14.0 N, CV = 8.8%, ICC = 0.947; Pallof press: SEM = 18.8, CV = 12.2%, ICC = 0.961). Student’s *t*-test revealed no significant differences between the test and retest measurements, either absolute (*p* > 0.418, ES < 0.10) or relative values (*p* > 0.314, ES < 0.15). Sensitivity analyses (Table 2) revealed differences between men and women in absolute force values; however, these differences were mitigated when comparing relative force values (N·bm^−1^). Bland–Altman plots showing the errors and 95% limits of agreement (LoA) between test and retest are depicted in Figure 3.

## 4. Discussion

The results of this study demonstrated that two submaximal, resistance-band tests (unilateral rows and Pallof press) are feasible and moderately reliable to evaluate the stability of trunk muscle strength in people with chronic low back pain. The use of portable force sensors allowed for providing real-time feedback and data collection. To the best of our knowledge, this is the first study examining a practical test to evaluate and monitor strength in people with chronic low back pain using resistance bands and a portable force gauge. Thus, following the current procedures and considering the errors of measurement, these tests could be incorporated into health centers or sports clubs to assist clinicians and strength and conditioning coaches (S&C) in programming tailored resistance training in people with chronic pain. In addition, these tests may improve home-based exercise programs in maintaining proper compliance, enhancing encouragement to exercise, and providing proper dosage of effort and feedback.

Previous studies have examined the reliability of isometric trunk muscle strength in people with and without low back pain using handheld [34] or isokinetic dynamometry [29]. Isokinetic dynamometers stand as the gold standard for testing trunk flexion and extension in this population (CV = 5 to 10%) [35] with handheld dynamometers remaining insufficient to achieve a similar accuracy [34]. Our novel approach using elastic bands provides a practical measure of trunk muscles’ stability comparable to isokinetic dynamometers in terms of reliability (CV < 10%), particularly when recording force values relative to body mass. The validity of elastic bands to examine isometric strength comparable to dynamometers has been proved early in healthy populations with excellent results (CV = 3 to 8%), for instance, in testing shoulder muscle strength [29] or knee flexion and extension strength [36]. These studies, together with our findings, reinforce the practical utility of elastic bands to test isometric force in both healthy and chronic low back pain populations without the need for expensive equipment. Notwithstanding the above, further research is needed in chronic low back populations to identify the safety and reliability of these and other testing conditions.

An important contribution of this study is the identification of expected errors from a given test. This information is useful as it allows coaches to determine whether the changes in performance after an intervention are due to strength improvements (i.e., when changes are higher than the test error) rather than a technical error [23,24]. According to our findings, one can expect errors below 20 N or 0.25 N·kg^−1^ when performing the unilateral row or Pallof press tests in people with chronic low back pain. In practical terms, clinicians or S&C coaches can identify true changes in performance by testing at the beginning of the exercise intervention and finding improvements over these errors. It is important to note that the current sample was not familiar with the procedures. However, this may prove that the current results can be used without the need for pre-testing sessions, thus saving time and is more customary in practice. Furthermore, because errors can be reduced after familiarization [23], it seems advisable to conduct several measurements during the training period to track performance.

A common limitation of using resistance bands is that one cannot be totally convinced about the training load as when using fixing loads like barbells, dumbbells, or machines. The current proposal suggests including a portable force sensor to monitor the tension of the band during each execution. Earlier practical guidelines have suggested the use of a pre-test using a force plate and a rack [30]; however, this approach requires specific equipment and does not allow to check the outcomes in each execution. The current novel approach using a portable force sensor allows overcoming this limitation and makes it possible to track performance in real time. Furthermore, considering that the resistance band may suffer from deterioration and loss of tension [37], the use of force sensors ensures that the patient is achieving the desired load using a more objective approach.

The type of exercises tested (unilateral row and Pallof press) is particularly effective in strengthening the trunk stability muscles. These exercises have been included in interventions for people with low back pain [38], hip surgery rehabilitation [39], or oncology population [40,41]. However, to the best of our knowledge, this is the first report showing data about the errors of measurement of a specific strength test for these exercises in people with low back pain. Other studies measured the changes in maximum isometric strength of the trunk flexors and extensors using a strain gauge dynamometer before and after lumbar fusion [42,43]. However, these results may be limited by a lack of understanding of the reliability of the procedures. Future studies should consider examining the errors of measurement of the tests being conducted for better data interpretation.

According to the last exercise prescription and progression considerations of the American College of Sports Medicine for people with chronic low back pain [44], patients should perform sets of 8–15 repetitions with 40%–70% 1RM intensity. Taking into account our novel approach to monitoring the training load in real time, it would be interesting to incorporate these technologies to have control of the training load, fatigue incurred, and strength adaptations. For example, if a person ends the test with 170 N, one can use this baseline as a 1RM value to prescribe tailored resistance training at a given submaximal intensity (e.g., 12 repetitions at 70% of the maximum load, 119 N). Future studies are encouraged to follow this approach and identify optimal training intensities for people with low back pain using the proposed submaximal resistance band test.

One common concern when using elastic bands is the progressive loss in the resistance provided as a result of their deterioration. To our knowledge, there is no accurate data to determine the lifespan of elastic bands. Based on our experience, resistance bands can last 6 to 8 months if using them daily and up to one year or two if using them twice a week. Traditionally, elastic bands are replaced when they show signs of degrading, delamination, perishing (cracking), rips, or tares. However, bands might reduce their resistance before these signs of deterioration are noticeable. One of the advantages of the technology-based approach we conducted is the knowledge of the actual resistance provided by the bands (i.e., Newtons). Thus, one can accurately identify when the equipment has become useless and should be replaced based on the amount of force provided before recording signs of deterioration.

This study may be limited due to the heterogeneity of the sample, involving people with different low back pain diagnoses, clinic history, fitness, and nutritional status. The use of different resistance bands may also alter the results. In addition, tests were not adapted to the participant’s height.

## 5. Conclusions

The present unilateral row and Pallof press procedures can be used as reliable screening tests for healthy adults and individuals with low strength levels (e.g., spinal injuries, oncology population, and older adults). Health professionals should incorporate the current submaximal, resistance-band tests (unilateral rows and Pallof press) and use force sensors to evaluate the stability of trunk muscle strength in people with chronic low back pain. Post-intervention changes over 20 N or 0.25 N·kg^−1^ should be considered to guarantee that true strength adaptations occurred. Although evaluators should apply dynamic strength assessments when possible, the extremely low strength level of some people (e.g., spinal injuries, oncology population, and older adults) would hinder or even preclude their implementation. These evaluations could be ideal alternatives to easily and reliably obtain strength values in these situations. This contribution may have an impact on improving the individualization and control of rehabilitation or physiotherapy and physical training in people with chronic low back pain.

## Figures and Tables

**Table 1:** Test–retest reliability for the two resistance band tests.

	M ± SD		SEM		CV (%)		ICC	
	Healthy	CLBP	Healthy	CLBP	Healthy	CLBP	Healthy	CLBP
**ALL SAMPLE**								
**Unilateral row**								
D Absolute force (N)	174 ± 41	161 ± 44	14.0	18.8	8.2	12.0	0.937	0.901
D Relative force (N·bm^−1^)	2.3 ± 0.4	2.0 ± 0.5	0.16	0.22	8.4	11.3	0.907	0.897
ND Absolute force (N)	175 ± 40	163 ± 44	12.5	14.0	7.2	8.8	0.948	0.947
ND Relative force (N·bm^−1^)	2.3 ± 0.4	2.0 ± 0.5	0.16	0.20	7.3	8.2	0.917	0.944
**Pallof press**								
D Absolute force (N)	167 ± 41	160 ± 46	17.8	12.9	10.8	8.4	0.899	0.960
D Relative force (N·bm^−1^)	2.2 ± 0.5	2.0 ± 0.5	0.25	0.16	11.6	8.4	0.870	0.952
ND Absolute force (N)	166 ± 42	160 ± 47	18.1	18.8	11.1	12.2	0.902	0.961
ND Relative force (N·bm^−1^)	2.2 ± 0.5	2.0 ± 0.5	0.25	0.16	11.9	8.4	0.846	0.949
**MEN**								
**Unilateral row**								
D Absolute force (N)	195 ± 23	184 ± 42	14.0	18.8	7.2	10.7	0.937	0.901
D Relative force (N·bm^−1^)	2.3 ± 0.4	2.2 ± 0.5	0.19	0.22	7.9	10.5	0.901	0.897
ND Absolute force (N)	196 ± 22	186 ± 43	12.5	14.0	6.4	7.8	0.948	0.947
ND Relative force (N·bm^−1^)	2.4 ± 0.4	2.2 ± 0.5	0.16	0.16	6.9	7.6	0.917	0.944
**Pallof press**								
D Absolute force (N)	187 ± 26	182 ± 41	17.8	12.9	9.5	7.4	0.899	0.960
D Relative force (N·bm^−1^)	2.3 ± 0.5	2.2 ± 0.4	0.25	0.16	11.0	7.8	0.870	0.952
ND Absolute force (N)	185 ± 26	180 ± 43	18.1	18.8	9.9	10.9	0.902	0.961
ND Relative force (N·bm^−1^)	2.2 ± 0.5	2.1 ± 0.4	0.25	0.16	11.4	7.8	0.846	0.949
**WOMEN**								
**Unilateral row**								
D Absolute force (N)	124 ± 27	133 ± 26	14.0	18.8	11.8	14.1	0.937	0.901
D Relative force (N·bm^−1^)	2.0 ± 0.5	1.8 ± 0.4	0.19	0.22	9.6	12.5	0.907	0.897
ND Absolute force (N)	125 ± 26	134 ± 22	12.5	14.0	10.4	10.5	0.948	0.947
ND Relative force (N·bm^−1^)	2.0 ± 0.4	1.8 ± 0.4	0.16	0.16	8.3	9.1	0.917	0.944
**Pallof press**								
D Absolute force (N)	119 ± 32	129 ± 36	17.8	12.9	15.6	10.4	0.899	0.960
D Relative force (N·bm^−1^)	1.9 ± 0.5	1.7 ± 0.5	0.25	0.16	13.4	9.6	0.870	0.952
ND Absolute force (N)	119 ± 36	130 ± 36	18.1	18.8	15.6	14.9	0.902	0.961
ND Relative force (N·bm^−1^)	1.9 ± 0.6	1.7 ± 0.6	0.25	0.16	13.4	9.5	0.846	0.949

Note: D, dominant hand/side; ND, non-dominant hand/side; CLBP, people with chronic low back pain; M, mean; SD, standard deviation; SEM, standard error of measurement; CV%, coefficient of variation; ICC, intraclass correlation coefficient.

**Table 2:** Sensitivity analysis between men and women.

	Healthy			CLBP		
	Mdiff (Error)	*p*	ES	Mdiff (Error)	*p*	ES
**Unilateral row**						
D Absolute force (N)	51 (16) *	0.006	1.43	70 (11) *	<0.001	3.06
D Relative force (N·bm^−1^)	0.4 (0.2)	0.077	0.85	0.3 (0.2)	0.160	0.75
ND Absolute force (N)	52 (16) *	0.006	1.44	70 (11) *	<0.001	3.15
ND Relative force (N·bm^−1^)	0.4 (0.2)	0.069	0.88	0.3 (0.2)	0.147	0.77
**Pallof press**						
D Absolute force (N)	18 (22)	0.427	0.38	67 (12) *	<0.001	2.64
D Relative force (N·bm^−1^)	0.1 (0.2)	0.564	0.27	0.3 (0.2)	0.233	0.63
ND Absolute force (N)	21 (22)	0.344	0.45	65 (13) *	<0.001	2.46
ND Relative force (N·bm^−1^)	0.1 (0.2)	0.438	0.37	0.2 (0.2)	0.264	0.59

Note: D, dominant hand/side; ND, non-dominant hand/side; CLBP, people with chronic low back pain; Mdiff, mean difference. * Significant differences between men and women (Student’s *t*-test, *p* < 0.05).

## Data Availability

Data are available upon request.
